# Development of mental disorders after removal of pituitary tumors

**DOI:** 10.1192/j.eurpsy.2026.10437

**Published:** 2026-07-31

**Authors:** Y. Sidneva, O. Zaitsev, S. Urakov, P. Kalinin, L. Astafyeva, A. Shkarubo, M. Kutin, I. Voronina, D. Fomichev, O. Sharipov, D. Andreev, I. Chernov, I. Klochkova, A. Donskoy, O. Rastvorova, D. Adueva

**Affiliations:** 1 neuropsychiatry, N.N.Burdenko National Medical Research Institute of Neurosurgery; 2 neurorehabilitation, Clinical and Research Institute of Emergency Pediatric Surgery and Trauma; 3 diagnostic; 4 neurosurgery; 5 neuroendocrinology; 6 neurology, N.N.Burdenko National Medical Research Institute of Neurosurgery, Moscow, Russian Federation

## Abstract

**Introduction:**

Pituitary tumors differ in histology, topographic and anatomical localization, hormonal activity. Clinical symptoms are represented by general cerebral disorders, neurological, visual, endocrine and mental. According to different researchers, the incidence of mental disorders ranges from 8 to 89%.

**Objectives:**

analysis of the development of mental disorders after removal of a pituitary tumor on a benign tumor model - craniopharyngioma (CP).

**Methods:**

132 patients over 18 years old: with endosuprasellar (29), stalk (48), extra-intra- (38) and intraventricular CP (17). Psychopathology was revealed in 86% of patients: with endosuprasellar localization, paroxysmal (48%) and asthenic (48%) disorders are characteristic; with suprasellar (stalk) - emotional (72%, including non-spontaneity 7%), amnestic (56%, including Korsakoff’s syndrome 2%) and paroxysmal (40%); with extra-intraventricular - amnestic (72%, including Korsakoff’s syndrome 36%), emotional-volitional (68%, including non-spontaneity 21%) and paroxysmal (50%); with intraventricular in the third ventricle - emotional-volitional (83%, of which non-spontaneity 8%), amnestic (83%, of which Korsakov’s syndrome 25%) and paroxysmal (50%). **Methods** used: the main clinical-psychopathological, also taken into account the data of examinations by a neurologist, endocrinologist, neuroimaging.

**Results:**

After removal of craniopharyngioma, mental disorders have the following development: without dynamics, decrease and increase. With endosuprasellar localization in the early postoperative period, the frequency of mental disorders decreased by 1.5-2 times; with suprasellar (stalk) localization - the number of patients with non-spontaneity (20%), Korsakoff’s syndrome (15%) increased, paroxysmal disorders were significantly less frequent after surgery (11%); with extra-intraventricular localization - the number of patients with Korsakoff’s syndrome (up to 50%), non-spontaneity (up to 30%) increased; with intraventricular localization - the incidence of non-spontaneity increased (up to 25%), paroxysmal symptoms decreased by 2 times. 78% of patients were discharged with mental disorders: in 23% at the preoperative level; in 12% a decrease; 43% had deterioration (of which Korsakoff’s syndrome 12%, non-spontaneity 8%, combination 13%).

**Conclusions:**

Mental disorders develop after removal of a pituitary tumor; the frequency of occurrence varies depending on the level of damage to the diencephalic region. More severe disorders are detected with suprasellar localization of the tumor and in the third ventricle (stalk, extra-intra- and intraventricular). In 43% of patients, worsening of psychopathology was detected after removal of a pituitary tumor.

**Disclosure of Interest:**

None Declared

